# Family fun or cultural free-for-all? A critique of the 2015 National Football League Super Bowl commercials

**DOI:** 10.15171/hpp.2016.06

**Published:** 2016-03-31

**Authors:** Corey H. Basch, William D Kernan, Rachel Reeves

**Affiliations:** Department of Public Health, William Paterson University, Wayne, NJ 07470, USA

**Keywords:** Super Bowl, Commercial, Advertising, Television, Violence, Risky behavior

## Abstract

**Background:** The purpose of this cross-sectional study was to enumerate and describe violent and risky behaviors as well as other general health behaviors exhibited in the advertisements during the National Football League (NFL) Super Bowl 2015.

**Methods:** Commercials during the NFL Super Bowl 2015 were assessed for violent and risky behaviors. Additional health behaviors were indicated such as the advertisement of unhealthy food, promotion of physical activity, and sexual content.

**Results: ** A total of 110 commercials were documented, accounting for 64 minutes of broadcast time. Commercials promoting automobiles, television shows, food, and movies were the most prevalent, representing just over half (53.7%) of all of the advertisements featured. Depictions of unsafe driving were found in 10.9% (n = 12) of the commercials. All 12 commercials contained some sort of risky or wild driving behavior, and speeding was observed in 11 of the 12 commercials. A total of 32 (29.1%) of the commercials were coded as including violent content.Physical activity behavior was present in 3 (2.7%) of the commercials. Conversely, substance use was observed in 3 (2.7%) of the commercials, none of which included health promotion messaging. Of the 110 commercials aired during the 2015 Super Bowl, 12.7% (n = 14) included sexual content.

**Conclusion: ** Parents should consider the possibility that their children may observe acts of violence or conflicting safety messages during commercial breaks.

## Introduction

 The National Football League (NFL) Super Bowl Championship is one of the most viewed television events in the United States, with an estimated 114.4 million viewers in 2015; up from 112.2 million in 2014.^1^ Previous studies on the content of television commercials aired during major sporting events have found that violent and age inappropriate advertisements are routinely aired during this period.^2,3^ The unpredictability of these commercials poses a challenge for parents who may want to reduce their child’s exposure to violent programming, especially when these sporting events are promoted as a means of family bonding.^2^ Naturally, because of the chance to reach so many viewers, advertising spots are coveted.

 It was estimated that the 2015 30-second Super Bowl ads were priced at $4.5 million each,^4^ a cost that reflects the importance of the event’s tremendous viewing numbers, and attracts large corporations with multimillion dollar marketing budgets. In fact, a company that promotes soda and snack food has spent $76.6 million on Super Bowl commercials from 2010 to 2014,^5^ during a time when concerns about childhood obesity are quickly rising.

 Researchers have demonstrated that the duration and content of children’s television viewing behaviors are directly related to their desire to eat foods low in nutrient density.^6^ One study found that non-nutritious food commercials had an immediate desirable effect on children who watched an average of 21 or more hours of television per week; a rate that is now considered slightly under normal for the average 12-17 years old (23:41 hours per week).^7^ Another study concluded that preschool-age children were three times more likely to express a desire for a food product that they saw an advertisement for twice within one hour of television viewing. The extent to which adults’ behaviors are influenced by commercials has also been explored.

 For example, research confirms that risky driving is depicted in driving commercials.^8,9^ In a study of 175 participants, it was determined that these types of commercials did not have an immediate effect on driving behavior of the participants.^10^ However, the authors suggested that further research is needed to determine the overall impact these depictions have on attitudes toward safe driving practices and cultural norms.^10^

 Previous studies have indicated that directly witnessing or becoming a victim of interpersonal violence as a child can lead to increased morbidity and mortality as a result of added stress and heightened inflammatory responses on the developing body.^11^ In addition, witnessing these actions has been shown to produce lower memory capacity later in life.^12^ Further research is needed to determine if the effects of witnessing violence in the virtual realm could also produce similar harmful consequences.

 There is a paucity of research on the content of commercials aired during major sporting events, with focus on the NFL Super Bowl being particularly limited. A literature search identified one older study conducted over a two year span (2001-2002) which analyzed of all major American competitive events identified the NFL Super Bowl as the event with the highest percentage of unsafe or violent commercials (48%).^3^ Sixty-five percent of those deemed violent or unsafe were advertising movies. Researchers have reported that motion picture advertisements aired during the NFL Super Bowl result in an overall 40% average increase in sales as compared to non-Super Bowl promoted films.^4^ Despite the large reach and the implications, content of NFL Super Bowl ads have rarely been analyzed from a public health perspective. Therefore, the purpose of this study was to enumerate and describe violent and risky behaviors as well as other general health behaviors exhibited in the advertisements during the NFL Super Bowl 2015.

## Materials and Methods

 Advertisements were recorded and analyzed by one researcher (RR) within the month following the February 1, 2015 broadcast. Each commercial was sorted by duration, broadcast reach, and type of product (food item, motor vehicle, etc). Short promotional inserts measuring less than 10 seconds were not included in the study. Each commercials hashtag, if applicable, was recorded and searched on the twitter analytics website topsy.com to determine the number of tweets sent in direct relation to the advertisements.^13^ Hashtag data was observed for the month following the event to observe the short term reach of each campaign. Two authors (CHB and RR) analyzed 10 videos independently to assess inter-rater reliability using Cohen’s kappa and was found to be 1.00, indicating perfect agreement.

 Commercials were assessed for violent and risky behaviors. Those deemed violent were categorized by the action depicted, and if advertising a motion picture, included the film’s rating. These included illustrations of physical aggression, often striking or using a weapon against another, or the use of explosives. Explanations of risky behaviors were indicated and most often included portrayals of erratic driving, speeding, weapon fighting, or substance use. Additional health behaviors were indicated, such as the advertisement of unhealthy food, promotion of physical activity, and presence of sexual content. If more than one health behaviors were present in an advertisement both were recorded and categorized accordingly. Several commercials were marked as portraying a conflicting or confusing message. These advertisements were interpreted to be promoting a feeling (love, acceptance, etc.) rather than the actual product in question, in addition to those ads that aired segments with an unrelated message.

 Descriptive analyses of the advertisement and content characteristics were completed using SPSS version 23. These analyses included calculating frequencies and percentages, means, and standard deviations.

## Results

 A total of 110 commercials aired during the 2015 Super Bowl comprised this sample, accounting for 3866 seconds (64 minutes) of broadcast time. Commercials aired during the Super Bowl promoted a wide variety of products and services, including consumable goods (food and beverages, automobiles), entertainment (movies, video games, vacations), and services (insurance, website hosting, phone service). Commercials promoting automobiles, television shows, food, and movies were the most prevalent, representing just over half (53.7%) of all of the advertisements featured. The type of service or product depicted in Super Bowl 2015 commercials are categorized in Table 1.

**Table 1:** Type of service/product depicted in Super Bowl 2015 commercials (n = 110)

	**n (%)**	**Average length (seconds)**
Automobile	18 (16.4)	48.2
TV show	18 (16.4)	21.1
Food	13 (11.8)	33.4
Movie	10 (9.1)	35.9
Insurance	8 (7.3)	27.9
Phone	6 (5.5)	27.5
Alcohol	5 (4.5)	50.8
Game	3 (2.7)	35.0
Website hosting	3 (2.7)	30.0
Auto accessory	2 (1.8)	30.0
Credit card	2 (1.8)	29.5
Microsoft	2 (1.8)	60.0
Party supplies	2 (1.8)	15.0
Vacation	2 (1.8)	45.0
Other	16 (14.5)	39.4
Total	110 (100)	31.5

 While the commercials consumed over an hour of broadcast time, there was great variability in their length, with the mean length at 31.5 seconds (min=10 seconds, max=90 seconds, range=80 seconds). When looking at categories that included three or more commercials, those that tended to run longer on average were those commercials that featured alcohol (x̄ = 50.8 seconds) and automobiles (x̄ = 48.2 seconds).

 Of the 110 Super Bowl 2015 commercials, 37.3% (n=41) contained a clear and direct health message. Of these 41 commercials, 10 (24.4%) contained a message deemed to be health promoting. These commercials consumed 493 seconds of broadcast time and had an average length of 49.3 seconds. The remaining 30 commercials contained content and/or images that conveyed negative health messaging spanning a total of 1124 seconds of airtime and averaging 40.1 seconds of broadcast time. Advertisements deemed containing negative health messages included depictions of violence, risky behaviors, unsafe driving, and messages promoting in non-nutritious food choices.

 As seen in Table 2, health messages were most prevalent in commercials containing content related to food (n=15; 13.6%). Of the 15 commercials with such content, only one commercial contained a health promoting message. The remaining 14 featured content that reflected or demonstrated poor eating habits and food choices. These advertisements most often included actors consuming high fat/low nutrient density fast food, prepackaged snack goods, and sugar sweetened beverages.

**Table 2:** Health messaging in Super Bowl 2015 commercials (n=110)

**Variables**	**No. of commercials with health message n (%)**	**No. of commercials with a health message that were health promoting n (%)**
Food	15 (13.6)	1 (6.6)
Unsafe driving	12 (10.9)	0 (0)
Physical activity	3 (2.7)	3 (100)
Substance use	3 (2.7)	0 (0)
Other	8 (7.3)	6 (75.0)
Total	41 (37.2)	10 (25.0)

 Depictions of unsafe driving were found in 10.9% (n = 12) of the commercials, none of which were coded as containing a health promotion message. As seen in Table 3, all 12 commercials contained some sort of risky or wild driving behavior, and speeding was observed in 11 of the 12 commercials. However, not all depictions of unsafe driving were found in automobile commercials. Of the 12 commercials depicting unsafe driving, only 58.3% (n=7) promoted the sale or lease of automobiles.

**Table 3:** Content of messaging related to unsafe driving practices in Super Bowl 2015 commercials (n = 110)

**Variables**	**n (%)**	**Average length**	**Results found in automobile Ads n (%)**
Risky/wild driving behavior	12 (10.9)	44.9	7 (58.3)
Speeding behavior	11 (10)	47.6	7 (63.6)

 As noted previously, the average length of automobile commercials (x̄ = 48.2 seconds) was higher than the average length of all commercials (x̄ = 31.5 seconds). Similarly, the average length of commercials depicting risky/wild driving (x̄ = 44.9 seconds) and speeding behavior (x̄ = 47.6 seconds) was also higher than the mean for all commercials.

 Substance use and physical activity, while observed less frequently than food and unsafe driving, were also health behaviors observed in multiple Super Bowl 2015 commercials. Physical activity behavior was present in 3 (2.7%) of the commercials, and all of these commercials contained positive, health promoting messages. Conversely, substance use was observed in 3 (2.7%) of the commercials, none of which included health promotion messaging (Table 2).

 While not considered health behaviors in this study, commercials were also coded for the presence of sexual content and violence. Of the 110 commercials aired during the 2015 Super Bowl, 12.7% (n=14) included sexual content. The average length of these commercials was 31.1 seconds, which is very close to the mean for all commercials (x̄ = 31.5 seconds) (Table 4).

**Table 4:** Length of messaging in Super Bowl 2015 commercials (n = 110)

**Variables**	**n**	**Average length (in seconds)**	**Total length (in seconds)**
All Super Bowl 2015 ads	110	31.5	3866
Ads with a Health Promotion Message	10	49.3	493
Ads with a Negative Health Message	28	40.1	1124
Ads Depicting Unsafe Driving	12	44.9	539
Sexual content	14	31.1	435
Ads Depicting Violent Behavior	32	34.2	1093

 A range of actions and behaviors coded as violence were observed in the 2015 Super Bowl commercials. Occurrences of violence ranged from minor instances, such as rough horseplay and bullying/intimidation, to messages that were life-threatening, including torture, firearm use, and murder/attempted murder. A total of 32 (29.1%) of the commercials were coded as including violent content. These commercials were slightly longer than the average at 34.2 seconds per commercial. Commercials with violent content consumed 28.3% of the total viewing time of the commercials aired (1093 of 3866 seconds) during the 2015 Super Bowl (Table 4).

 One fourth of all Super Bowl commercials (28/110) offered a hashtag for discussion on social media. In the month following the 2015 Super Bowl, 1 017 526 tweets were sent that referred to individual commercials and brands advertised during the event. The average number of tweets was 36 340 and they ranged from 229-380 614. The most tweeted commercial (380 614 tweets) represented a social marketing campaign to end sexism.

## Discussion

 This study offers insight into the content of widely viewed commercials, which are relevant to public health. There is widespread agreement that advertising can have a tremendous negative effect on societal norms, child development, and levels of self-esteem among viewers.^14^ The American Academy of Pediatrics has identified a strong causal relationship between violent media viewing and aggressive behavior among children.^15^ While these studies have traditionally focused on full-length programs like television shows and movies, there is a paucity of research on the influence that these short violent images have on viewers, especially impressionable children.

 Nearly 30% of the recorded 2015 Super Bowl commercials depicted acts of violence, of which, included commercials displaying gunshots, sword fighting, visible bloodshed, and intimidation. While censoring commercials is possible, it can prove to be difficult. For instance, although parents may be able to shield their children from seeing a violent film advertised in these commercials, they are ultimately unable to prevent them from witnessing the short 30 second images of intense violence that epitomize the action movie genre.

 Unsafe driving practices were observed in 11% of the 2015 Super Bowl Commercials. These practices included speeding, aggressive driving, and maneuvers performed by stunt drivers. Among young adults in the United States, motor vehicle accidents rank as a leading cause of death, and it is well documented that unsafe driving practices contribute to crashes.^8^

 The Federal Communications Commission does not currently regulate levels of violence in network television advertising^16^ like the Alcohol and Tobacco Tax and Trade Bureau (TTB) does.^17^ Under the TTB, alcoholic beverage companies can air television advertisements during programming that can historically demonstrate that no more than 30% of the viewing audience is under the age of 21.^17^ The 2012 Super Bowl audience was estimated to be comprised of 39% viewers under the age of 34.^18^ Nielsen has estimated that 66% of all 18-21 year olds in the United States tuned in to watch the 2014 Super bowl.^19^ Additionally, a survey conducted by the National Retail Foundation found that one in five 18-24 year olds specifically find the commercials to be the best part of the Super Bowl game day experience.^20^

 Nearly 5% of the commercials aired during the 2015 Super Bowl promoted the sale and consumption of alcoholic beverages. Researchers have determined that alcohol advertisements have a profound effect on the number and frequency of underage drinking occurrences in the United States.^21^ Between 2001 and 2009, exposure to alcohol related marketing increased by 71% among underage TV viewers.^22^ Because a positive relationship between ad consumption and the likelihood of initiating underage drinking has been identified,^21^ the increase in marketing could reasonably be considered a contributing factor to the rise in underage drinking. Additionally, it has been reported that 4750 children under the age of 16 try alcohol for the first time each day in the United States.^22^ Underage drinkers are estimated to account for 11%-20% of the entire US alcohol market.^23^

 Researchers have pointed to the frequent display of lavish material items and unattainable body images in advertising as a main contributor to the internalized feelings of inadequacy that commonly arise among highly consumer driven cultures.^14^ Commercials that romanticize material items and attempt to promote feelings of inclusion based on tangible goods can often lead people to feel inadequate if they do not own said item.^14^ Additionally, commercials will frequently use conventional gender differences to evoke laughter, perpetuating the cycle of gender stereotypes.^24^

 Social media is becoming an increasingly common outlet for discussing commercials aired during the Super Bowl.^25^ The most tweeted commercial (380 614 tweets) represented a social marketing campaign to end sexism, suggesting that health promotion agendas could benefit greatly from the ability to begin instant and meaningful conversations on social media.

 This study was limited in the fact that the actual reach of the conveyed messages is most likely underestimated. Because the NFL Super Bowl is seen as a national event, many people host parties or frequent bars during broadcast to celebrate with friends; patrons who are not captured in viewing statistics. Additionally, this was a cross-sectional study, and thus is limited by collecting data at a single point in time.

 Although the Super Bowl is often seen as a family-friendly program, advertisements aired during the event can often be unsuitable for young eyes. Parents should consider the possibility that their children may observe acts of violence or conflicting safety messages during these short unregulated periods during broadcast.

## Ethical approval

 The IRB at William Paterson University does not review studies that do not include human subjects.

## Competing interests

 The authors have no conflicts of interest to declare.
